# NLRP3 Inflammasome and Allergic Contact Dermatitis: A Connection to Demystify

**DOI:** 10.3390/pharmaceutics12090867

**Published:** 2020-09-11

**Authors:** Ana Isabel Sebastião, Isabel Ferreira, Gonçalo Brites, Ana Silva, Bruno Miguel Neves, Maria Teresa Cruz

**Affiliations:** 1 Faculty of Pharmacy, University of Coimbra, 3000-548 Coimbra, Portugal; anaisabelsebastiao@gmail.com (A.I.S.); isabel.ferreira@ff.uc.pt (I.F.); g.sousabrites3@gmail.com (G.B.); 2 Center for Neuroscience and Cell Biology, University of Coimbra, 3004-504 Coimbra, Portugal; anacrs@cnc.uc.pt; 3 Department of Medical Sciences and Institute of Biomedicine—iBiMED, University of Aveiro, 3810-193 Aveiro, Portugal; bruno.neves@ua.pt

**Keywords:** allergic contact dermatitis, skin sensitizers, DAMPs, inflammasome, NLRP3

## Abstract

Allergic contact dermatitis is a common occupational disease that manifests as a cell-mediated hypersensitivity reaction following skin exposure to small reactive chemicals termed haptens. Haptens penetrate the stratum corneum and covalently modify proteins in the epidermis, inducing intracellular stress, which further leads to the release of damage-associated molecular patterns (DAMPs), such as uric acid, reactive oxygen species, hyaluronic acid fragments and extracellular adenosine triphosphate (ATP). These DAMPs are recognized by pattern recognition receptors (PRRs) in innate immune cells, namely dendritic cells (DCs), leading to their maturation and migration to the draining lymph nodes where they activate naïve T lymphocytes. Among all PRRs, several studies emphasize the role of NOD-, LRR- and pyrin domain-containing protein 3 (NLRP3) inflammasome on the allergic contact dermatitis (ACD) sensitization phase. However, skin allergens—danger signals—NLRP3 inflammasome axis is yet to be completely elucidated. Therefore, in this review, we sought to discuss the molecular mechanisms underlying DAMPs release and NLRP3 inflammasome activation triggered by skin allergens. The elucidation of these key events might help to identify novel therapeutic strategies for ACD, as well as the development of nonanimal alternative methods for the identification and potency categorization of skin sensitizers.

## 1. Introduction

**Figure 1:**
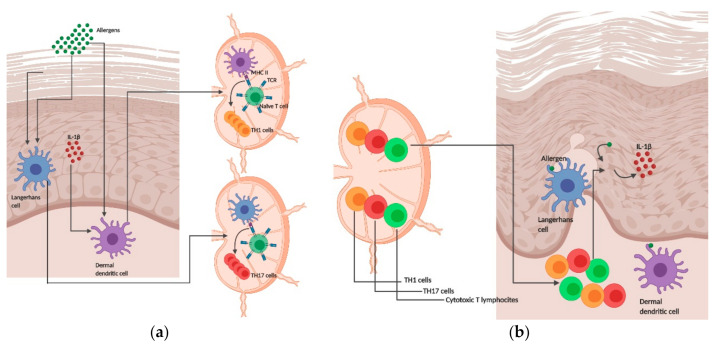
Schematic overview of the mechanisms underpinning skin sensitization during sensitization (**a**) and elicitation (**b**) phases: (**a**) Allergens penetrate the skin and are captured and processed by antigen-presenting cells (APCs), Langerhans cells (LC) and dermal dendritic cells (DCs). Concomitantly, the allergen promotes the release of interleukin (IL)-1β and other proinflammatory cytokines, contributing to the amplification of the immune reaction. Then, LC and DC leave the skin and migrate to skin-draining lymph nodes where they present the antigen to naïve T cells—priming, through the interaction of the major histocompatibility complex (MHC) II with T cell receptor (TCR) complex. This interaction triggers naïve T cells proliferation and differentiation into CD4^+^ and CD8^+^ cells. CD4^+^ and CD8^+^ T cells can further differentiate into other cell types, depending on the APC evoking the differentiation. (**b**) Upon re-exposure to the same allergen the differentiated cells migrate to the skin where they exert distinct functions. For example, cytotoxic T lymphocytes promote the apoptosis of keratinocytes expressing IL-1β and other proinflammatory cytokines. In parallel, CD4^+^ regulatory T cells migrate to the challenge site and promote inhibition of allergic contact dermatitis (ACD) and tolerance. Created with BioRender.com.

Allergic contact dermatitis (ACD) is one of the most common occupational diseases, affecting nearly 20% of the European population [1,2,3]. ACD is a type IV hypersensitivity response caused by repetitive exposure to a chemical sensitizer. These sensitizers are usually low-molecular-weight chemicals or metals (LMW), that are unable to elicit an immune response per se. Hence, LMW must covalently modify skin proteins or bind directly to major histocompatibility complex (MHC) molecules on the surface of antigen-presenting cells (APC) [4]. The pathophysiology of ACD is characterized by two distinct phases (Figure 1). First, the sensitization phase, when a susceptible individual is exposed to sensitizing chemicals, leading to the priming of the immune system. Second, the elicitation phase when re-exposure of sensitized individuals to the same chemical leads to ACD clinical manifestation within 24/72 h. These chemicals are highly electrophilic and can cross the stratum corneum, gaining access to viable epidermis [2,4], where they covalently bind to extracellular and cellular proteins—haptenization. These protein–hapten conjugates promote intracellular stress and xenoinflammation on epidermal cells, which is characterized by the release of damage-associated molecular patterns (DAMPs), such as reactive oxygen species (ROS), uric acid (UA), hyaluronic acid (HA) fragments and adenosine triphosphate (ATP) [5,6,7,8]. These DAMPs are then recognized by pattern recognition receptors (PRRs) such as Toll-like receptors (TLRs) and NOD-like receptors (NLRs) in innate immune cells, namely dendritic cells (DCs), leading to their maturation [9,10]. Upon capture and processing of the immunogenic hapten–protein complexes, dermal DCs undergo maturation and acquire the capacity to migrate toward local lymph nodes where they prime naive CD4^+^ and CD8^+^ lymphocytes. Naïve T cells become activated and differentiate into allergen-specific effector cytotoxic T lymphocytes (CTL) and effector T helper (T_H_) cells T_H_1 and T_H_17 (Figure 1a), which then disseminate systemically to peripheral tissues, including the skin (Figure 1b). At this point, immunological memory is mounted and the sensitization phase concluded [2,4].

Upon re-exposure to the same chemical, resident allergen-specific T cells and more effector cells are recruited into the skin where they promote a strong cutaneous inflammatory reaction, characterized by erythema, edema and vesiculation, leading to dryness, fissure and lichenification of the affected areas [11]. Although the studies addressing the role of APC on ACD are mainly focused on DCs, macrophages are also reported to play an important role. Indeed, macrophages were shown to be involved in DCs clustering in the perivascular area through CXCL2 signaling [12]. Furthermore, studies by Tuckerman, showed that corticosteroids (usually used for ACD treatment) ameliorate contact hypersensitivity (CHS) through therapeutic action on macrophages and neutrophils [13]. Surprisingly and although M2 macrophages play key roles in the suppression of T_H_1 cell response and the orchestration of tissue repair, they may exacerbate inflammation in some pathological conditions. Indeed, upon the hapten-induced CHS animal model of ACD, M2 macrophages accumulate in the challenging sites and produce matrix metalloproteinase 12 (MMP12), contributing to exacerbated CHS [14].

The capacity of chemical allergens to cause sterile inflammation has been demonstrated by several studies using animal models of CHS. In germ-free mice, exposure to contact allergens leads to the activation of PRRs involved in anti-microbial defense, suggesting that endogenous DAMPs play an important role in the development of ACD. Indeed, these danger signals are capable of activating various cell types from both innate and adaptive immune responses [15]. Several studies have shown the TLRs’ involvement in ACD, proving that they can recognize both DAMPs and allergens, thus contributing to the development of this inflammatory disease [16,17]. Watanabe and coworkers showed that NOD-like receptor family pyrin domain-containing 3 (NLRP3) inflammasome is present in keratinocytes and could be activated upon exposure to the extreme sensitizer trinitro-chlorobenzene (TNCB). Moreover, the authors showed impairment of CHS to TNCB and 1-fluoro-2,4-dinitrobenzene (DNFB) in mice lacking apoptosis-associated speck-like protein containing a caspase recruitment domain (ASC) and NLRP3, thus identifying NLRP3 inflammasome as a key regulator of the immune response in CHS [18].

**Figure 2:**
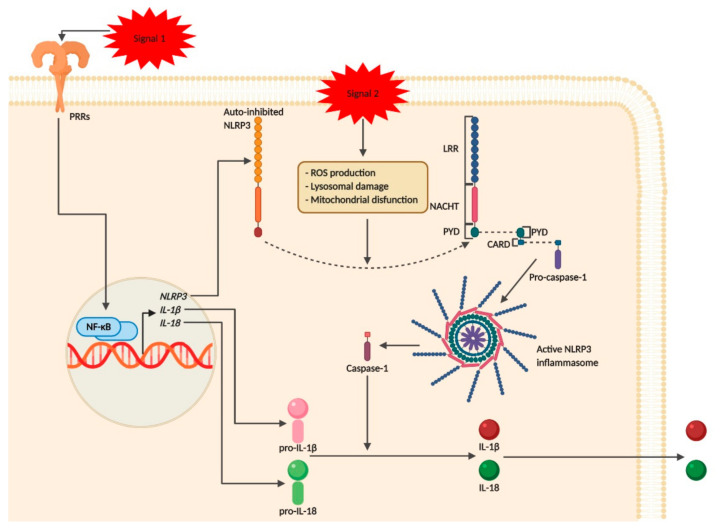
Pyrin domain-containing protein 3 (NLRP3) inflammasome activation. NLRP3 inflammasome is composed of a sensor molecule (e.g., NLRP3), the adaptor protein ASC and the cysteine protease pro-caspase-1. ASC binds NLRP3 and caspase-1 by pyrin domain (PYD) and caspase recruitment domain (CARD) domains, respectively. Before stimulation, NLRP3 is held inactive by post-translational modifications such as phosphorylation and ubiquitination. This multimeric protein platform is then activated by a two-step process: first, pattern recognition receptor (PRR) engagement (e.g. Toll-like receptors (TLRs)) through damage-associated molecular patterns (DAMPs) or pathogen-associated molecular patterns leads to NF-κB activation, resulting in increased expression of NLRP3, as well as other key proinflammatory genes, such as *IL1β* and *IL18* (Signal 1, priming step). Next, indirect activation of NLRP3 occurs by a plethora of signals (ROS, ion or membrane perturbations, or extracellular adenosine triphosphate (ATP)), resulting in ASC and pro-caspase-1 recruitment and subsequent NLRP3 inflammasome activation. After NLRP3 inflammasome activation, pro-caspase-1 turns into caspase-1 (which is the enzyme active form) and converts pro-IL-1β and pro-IL-18 into their active state. Created with BioRender.com.

Inflammasomes are cytosolic multiprotein signaling platforms that assemble in the presence of pathogenic microorganisms and sterile stressors and are involved in the control of the inflammatory response and coordination of antimicrobial host defense [19]. They are responsible for mediating caspase-1 activation (which promotes the secretion of the proinflammatory cytokines interleukin (IL)-1β and IL-18) as well as pyroptosis, a form of cell death induced by bacterial pathogens [20]. Structurally, inflammasomes typically comprise a sensor molecule connected to caspase-1 via ASC [21]. The adaptor protein ASC consists of two death-fold domains: a pyrin domain (PYD) and a caspase recruitment domain (CARD), which interacts with PYD and CARD domains of NLRs and pro-caspase-1, respectively [20]. Inflammasome assembly ultimately results in pro-caspase-1 dimerization, which is then followed by autocatalysis and activation of pro-caspase-1. Once activated, caspase-1 is responsible for processing pro-IL-1β and pro-IL-18 into their mature secreted forms (Figure 2) [22].

Inflammasome assembly can be activated by several inflammasome sensor molecules including the NLRs, retinoic acid-inducible gene 1 (RIG-1)-like receptors (RLRs), and absent in melanoma 2 (AIM2)-like receptors (ALR), although, the most comprehensively studied is NLRP3 inflammasome [9,10]. A two-signal hypothesis was proposed to explain NLRP3 inflammasome activation. First, the initial priming signal, provided by TLR engagement and subsequent activation of nuclear factor kappa-light-chain-enhancer of activated B cells (NF-κB), leads to the expression of inflammasome components and cytokines precursors, including pro-IL-1β and pro-IL-18. Second, an activation signal, prompted by endogenous sterile DAMPs, which triggers NLRP3 inflammasome assembly (Figure 2). These signals include intracellular calcium fluxes, potassium efflux, ATP, UA, mitochondrial DNA (mtDNA), extracellular matrix (ECM) compounds (such as LMW hyaluronan), protein kinase R (PKR) activation, ROS and release of contents from phagolysosomes upon frustrated phagocytosis of silica, asbestos, aluminum salts, amyloid deposits and cholesterol crystals (Figure 2) [5,7,20]. Before inflammasome activation, NLRP3 is held in its inactive form through a series of post-translational modifications such as phosphorylation and ubiquitination [9].

Interestingly, inflammasome activation has been suggested to be involved in a plethora of immune-related skin diseases, including CHS, cryopyrin-associated periodic syndrome psoriasis, vitiligo, systemic lupus erythematosus and atopic dermatitis. NLRP1 (caspase 1 negative regulator) polymorphisms are associated with an increased risk of developing psoriasis, vitiligo and lupus erythematosus. NLRP3 inflammasome expression was also reported to be increased in systemic sclerosis, acne and rosacea skin conditions, compared to healthy individuals [23]. Indeed, IL-1β and IL-18 play key roles in skin disorders, and several therapeutic drugs have been developed to target inflammasome activation as well as IL-1β signaling. Accordingly, patients with pustular psoriasis achieved remission upon treatment with an anti-IL-1β monoclonal antibody. Furthermore, P2X7 has been shown to attenuate murine lupus symptoms by inhibiting the activation of the NLRP3 inflammasome [24].

In this review, we sought to revise the molecular mechanisms underlying DAMPs release and NLRP3 inflammasome activation triggered by skin allergens, in the context of ACD. Although the involvement of inflammasomes in ACD has been extensively reviewed, data compilation and revision about the different DAMPs elicited by the most studied skin sensitizers and their involvement in inflammasome activation is still missing. Indeed, a deeper understanding of these events might help to define novel therapeutic strategies for ACD and to design novel nonanimal in vitro tests for the identification of sensitizers.

## 2. Skin Sensitizers and Danger Signals

The ability of skin allergens to induce sensitization relies on two main properties: (1) their high reactivity, which allows them to covalently bind proteins (carrier molecule) and therefore becoming immunogenic and (2) their proinflammatory properties and ability to induce the release of DAMPs, which are essential for the recruitment, migration and maturation of DCs [25]. Keratinocytes play an important role in ACD since they release chemokines and proinflammatory cytokines that are involved in DCs activation [26]. IL-1β is a proinflammatory cytokine that has been shown to play a central role in ACD [18,27,28]. It is first synthesized as a precursor protein (pro-IL-1β), which has to be proteolytically cleaved to a shorter, active molecule to be secreted. The generation of biologically active IL-1β is highly regulated and relies on inflammasome activation.

Indeed, IL-1β has a crucial role in DC function as well as in ACD. Cumberbatch and colleagues showed that, like tumor necrosis factor (TNF)-α, IL-1β is involved in DC accumulation in draining lymph nodes after skin sensitization. Furthermore, TNF-α and IL-1β were shown to induce the expression of adhesion molecules in LC and to promote LC ability to interact with and pass through the basement membrane [29]. More recently Nishibu et al. also reported that the IL-1β produced by keratinocytes, as well as TNF-α, are equally involved in the motile activities of epidermal LCs [30].

The most common inflammasome-triggering danger signals encompass UA, ATP, mtDNA, ROS and ECM compounds such as low-molecular-weight hyaluronic acid (LMWHA) [7,31].

### 2.1. Reactive Oxygen Species

**Figure 3:**
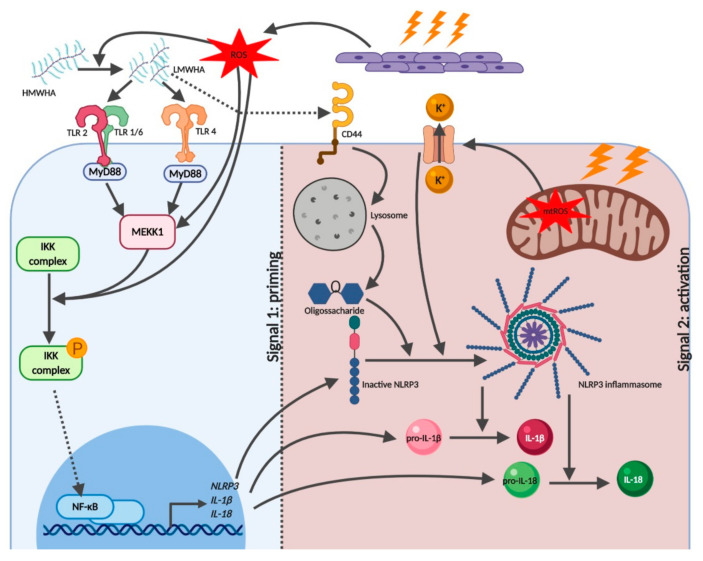
ROS and low-molecular-weight hyaluronic acid (LMWHA) involvement in both priming and activation of the NLRP3 inflammasome. ROS are rapidly produced in the skin following contact sensitizer treatment. ROS are responsible for triggering several intracellular cascades as well as promoting HA degradation. High-molecular-weight HA (HMWHA) is degraded into LMWHA, which is recognized by TLR2 and TLR4. Both TLR2- and TLR4- mediated signaling, as well as ROS, activate the NF-κB pathway, leading to *IL1β*, *IL18* and *NLRP3* transcription. Some allergens also induce mtROS production which leads to K^+^ efflux and NLRP3 inflammasome activation. LMWHA can activate NLRP3 inflammasome through CD44 (expressed on the surface of macrophages). CD44-LMWHA complex is further internalized and degraded by endosomes/lysosomes and the released oligosaccharides induce NLRP3 inflammasome activation. CD44, cluster of differentiation 44. Created with BioRender.com.

ROS are key signaling molecules involved in a myriad of physiological and pathophysiological processes. In ACD, ROS are responsible for amplifying the immune response through DC activation [32]. Ferreira and colleagues showed that the extreme sensitizer DNFB induces glutathione depletion, which in turn leads to increased levels of intracellular ROS together with p38 mitogen-activated protein kinase (MAPK) and c-Jun N-terminal kinase (JNK) activation [33]. JNK and p38 MAPK belong to the MAPKs family and are activated by MAPK kinase (MAP3 kinases) MEKK1 and ASK1, respectively [34]. MEKK1, as well as ROS, promote IκB kinase (IKK) phosphorylation and downstream NF-κB pathway activation [35,36]. Additionally, the NF-κB pathway can be activated by p38α protein isoform Exip [31]. NF-κB acts as a central regulator of innate immunity, controlling the expression of multiple immune and stress response effector molecules such as IL-1β and NLRP3 (Figure 3) [9,10,37]. Proinflammatory cytokines expression is also modulated by the activation of activator protein 1 (AP-1), which can be evoked by p38 MAPK and JNK [35]. More recently, it was shown that glucose-6-phosphate dehydrogenase (G6PD)-deficient cells display defective inflammasome activation and bacterial clearance due to impaired NADPH oxidase/p38 MAPK/AP-1 signaling [38]. Taken together, these results support the role of ROS in the priming step and assembly of the NLRP3 inflammasome.

Chromium is a widely known ACD-triggering agent. The naturally occurring valence of this metal, the trivalent form (Cr (III)), has lower dermal toxicity compared to the hexavalent form (Cr (VI)). In general, the Cr (VI) diffuses through the skin at a much higher rate. After skin penetration, Cr (VI) is reduced into Cr (III), which is then recognized by skin immune cells, triggering the immunological response of ACD [39]. This reaction leads to ROS production [40], which can affect redox-sensitive protein kinases and transcription factors. Indeed, Cr (VI) was shown to cause mitochondrial ROS (mtROS) accumulation in the monocytic cell line THP-1, causing K^+^ efflux and subsequent NLRP3 inflammasome activation [41]. Wang and colleagues also reported an increased ROS production in keratinocytes treated with Cr (VI), as well as increased activation of Akt, NF-κB and MAPK pathways, transcription of TNF and the release of IL-1α [28].

Nickel is another common metal that causes ACD [42]. Contrarily to Cr (VI), nickel binds to TLR4 and induces a TLR4-dependent activation of the signaling pathways involved in the NLRP3 priming step [42,43]. Additionally, nickel promotes second signals such as accumulation of mtROS, and cation fluxes that trigger NLRP3 assembly [43,44]. This is in agreement with the previously reported connection between mtROS and NLRP3 inflammasome activation (Figure 3) [9,10,37].

Common metals such as copper, cobalt, mercury and zinc also induce ROS production, although an association between ROS production by these compounds and inflammasome activation remains yet to be described [45,46,47,48].

### 2.2. Low Molecular Weight Hyaluronic Acid

The skin ECM is composed of a plethora of molecules such as collagen, elastin, glycoproteins and glycosaminoglycans [49,50]. One of its major components is HA, which belongs to the glycosaminoglycans family. HA has a key position in dermis metabolism, also playing an important role in wound healing and tissue repair processes. This is mainly due to its ability to retain water, maintaining an environment favorable to healing, and by its capacity to stimulate growth factors, cellular constituents and the migration of different cells, essential for skin regeneration [51]. There are two types of HA with different biological activities: high-molecular-weight HA (HMWHA) and LMWHA. HMWHA and LMWHA have immunosuppressive and proinflammatory properties, respectively. According to their length, LMWHA fragments have distinct proinflammatory properties. Indeed, LMWHA ranging from four to six saccharides were reported to activate the NF-kB signaling pathway with consequent cytokine production in DCs [52].

In an inflammatory environment, HMWHA is broken into LMWHA that further contributes to the amplification of the inflammatory response [53]. Therefore, LMWHA fragments act as endogenous DAMPs by binding and activating TLR2 and TLR4-dependent intracellular signaling cascades [54,55]. In the context of ACD, the treatment of keratinocytes with phenylenediamine (PPD) and 2,4-dinitrochlorobenzene (DNCB), increased the synthesis of HA through the induction of Hyaluronan Synthases (HS) 1 and 2, resulting in the formation of proinflammatory LMWHA fragments that interacted with TLR4 and activated the NF-κB signaling axis [55,56]. DNCB-induced LMWHA formation can also occur via ROS production [57]. Accordingly, several in vivo studies show that blocking ROS production and HA degradation (by antioxidants or hyaluronidase-inhibitors) prevents the sensitization and elicitation phases, in mice models of CHS [54]. These findings were later corroborated by Muto and colleagues who demonstrated that HA breakdown is an initiator of DC migration from the skin to lymph nodes following sensitization with a topical antigen (Figure 3) [58].

As stated above, LMWHA interacts with TLR4 and activates the NF-κB-regulated transcription of proinflammatory genes coding for pro-IL-1β, pro-IL-18 and NLRP3. Therefore, we can establish a link between sensitizers-induced LMWHA formation and the priming phase of the NLRP3 inflammasome (Figure 3) [9,10,37]. Yamasaki and colleagues demonstrated that HA-induced IL-1β secretion was dependent on NLRP3 activation. Indeed, mice lacking NLRP3 showed impaired IL-1β secretion in response to HA. The authors showed that HMWHA binds to the cluster of differentiation 44 (CD44) on the surface of macrophages, leading to NLRP3 inflammasome activation. This activation occurs through endocytosis of the CD44-HMWHA complex, followed by the endosomal/lysosomal degradation of the HMWHA into small oligosaccharide fragments (Figure 3) [59]. Since CD44 is present in keratinocytes [60] it is reasonable to speculate that sensitizer-induced LMWHA formation may trigger NLRP3 assembly by the same mechanism.

Given the role of LMWHA on the activation of crucial immunomodulatory pathways, expression of proinflammatory genes, as well as cytokines, it may be linked both to priming and assembly of the NLRP3 inflammasome.

### 2.3. Adenosine Triphosphate

**Figure 4:**
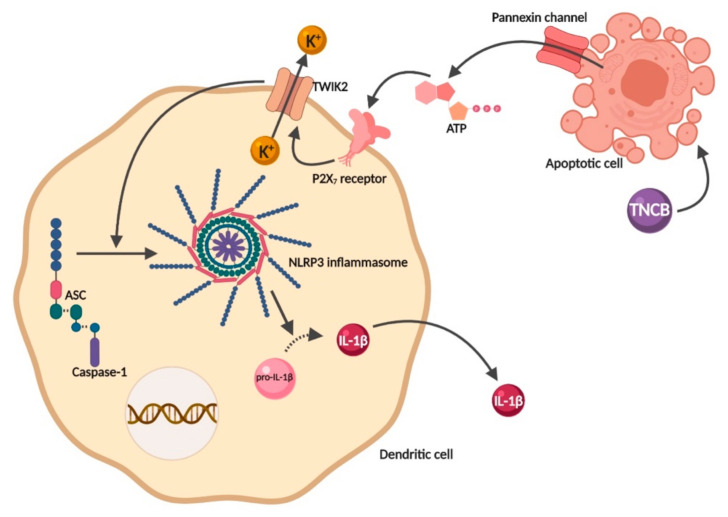
Trinitro-chlorobenzene (TNCB)-induced NLRP3 inflammasome activation via K^+^ channel after ATP release. Upon contact with the skin sensitizer TNCB, keratinocytes release ATP through the pannexin channel, which then binds to the P2X7 receptor in the cell surface. Once activated, the P2X7 receptor induces K^+^ efflux through TWIK2, leading to a reduction in intracellular K^+^ levels which activates the NLRP3 inflammasome. Once activated, inflammasome induces the maturation and secretion of IL-1β. Created with BioRender.com.

Apart from its commonly known role as the “molecular currency” for energy transfer within the cell, ATP also acts as a DAMP [61]. Upon allergen contact with the skin, keratinocytes and apoptotic cells release ATP into the extracellular milieu through pannexin channels, where it can bind to P2X7 receptors in DCs cell surface [62]. P2X7 is a ligand-gated ion channel that, upon activation, induces K^+^ efflux through the two-pore domain weak inwardly rectifying K^+^ channel 2 (TWIK2). Lower intracellular K^+^ leads to NLRP3 inflammasome activation and IL-1β secretion (Figure 4) [63,64].

Weber and colleagues observed ATP release in the skin upon TNCB treatment, indicating indirect contact allergen–dependent P2X7 triggering (Figure 4). Moreover, they showed, by cell transfer experiments, that P2X7 expression on DCs is crucial for sensitization, but not for the effector phase of CHS [64]. Interestingly, our group demonstrated no increase in P2X7 mRNA after treatment with DNFB [65].

Supporting the relevance of ATP in ACD pathophysiology, several studies showed that impaired ATP degradation regulates dermal DC motility, potentiating the sensitization phase induced by TNCB [62,66]. Moreover, P2X7-deficient DCs fail to induce sensitization to contact allergens and do not release IL-1β in response to LPS and ATP. Interestingly, the blockade of the P2X7 receptor by antagonist antibodies leads to a decrease in IL-1β secretion, resulting in a controlled inflammatory response in ACD mice models [67]. Therefore, modulation of the ATP-P2X7-NLRP3 axis may represent a promising strategy for the prevention or treatment of ACD.

### 2.4. Mitochondrial DNA

**Figure 5:**
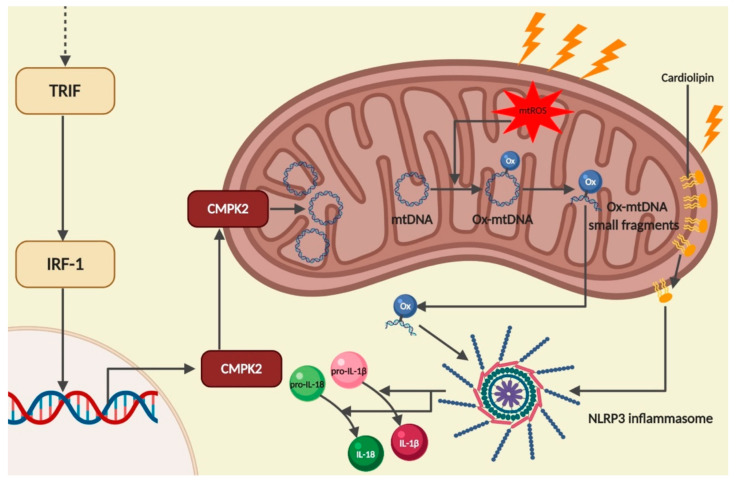
NLRP3 inflammasome activation via mitochondrial stress. After TLR4 recognition of a ligand, two main pathways are activated: the NF-κB pathway in an early phase, via MyD88 (not shown), and the IRF1, via TRIF. IRF1 triggers the transcription of CMPK2, a rate-limiting enzyme that supplies deoxyribonucleotides for mtDNA synthesis. CMPK2 translocates to the mitochondria, where it is involved in mtDNA synthesis. Upon mitochondrial stress, mitochondria produce ROS, which promote mtDNA oxidation. Ox-mtDNA is then broken into small fragments that are released into the cytosol, where they potentiate the activation of the NLRP3 inflammasome leading to more conversion of pro-IL-1β in IL-1β. Simultaneously, cardiolipin moves from the inner mitochondrial membrane to the outer surface where it binds to the LRR domain (not shown) and activates the NLRP3 inflammasome activity. Created with BioRender.com.

Mitochondrial DNA is a circular double-stranded molecule that in normal conditions is found on the mitochondrial matrix. However, in response to dysfunction or damage, mitochondria components are released or exposed, which triggers an immune response [68]. NLRP3 activators such as plasma membrane rupture, K^+^ efflux and increased intracellular Ca^2+^ levels lead to an increase in mtROS production and the release of mtDNA from mitochondria. In an oxidative microenvironment mtDNA is oxidized (ox-mtDNA) and associates with NLRP3, enabling NLRP3 inflammasome activation [69,70] (Figure 5). Strikingly, mitochondrial damage per se is not sufficient to trigger NLRP3 activation if priming stimulus is omitted [21].

Recently, Zhong and collaborators added a new piece of knowledge to this intricate network formed by TLR, ROS, mtDNA and inflammasomes. The authors showed that NLRP3 inflammasome priming and activation are linked through the induction of new mtDNA synthesis. Briefly, after TLR4 engagement, myeloid differentiation primary response 88—MyD88 (early time points) and TIR-domain-containing adapter—inducing interferon-β–TRIF (later stages) activate interferon regulatory factor 1 (IRF1)-dependent transcription of cytidine/uridine monophosphate kinase 2 (CMPK2), a rate-limiting enzyme that supplies deoxyribonucleotides for mtDNA synthesis [71].

Regarding the role of mtDNA release in ACD, it was shown that nickel, besides activating TLR4, also induces the cytosolic accumulation of both mtROS and mtDNA, contributing to IL-1β and IL-18 secretion via NLRP3 inflammasome activation [43].

### 2.5. Cardiolipin

The phospholipid cardiolipin is an essential constituent of mitochondrial membranes and plays a role in many mitochondrial functions, such as respiration and energy conversion. Cardiolipin is almost exclusively located in the inner mitochondrial membrane. However, upon mitochondria destabilization, cardiolipin is exposed on the mitochondria outer surface, where it can activate NLRP3 inflammasome via LRR [68,72,73].

Although studies addressing the role of cardiolipin in ACD remain limited, we previously reported that THP-1 cells treated with the skin sensitizer DNFB showed a striking increase in cardiolipins and that their remodeling may be an important mechanism by which allergens cause redox imbalance [74]. During inflammasome activation, NLRP3 associates with mitochondria; however, the role of this interaction remains undisclosed. Indeed, NLRP3 and caspase-1 independently interact with the mitochondrial lipid cardiolipin, which is externalized to the outer mitochondrial membrane at the priming step in response to ROS. After an activation signal, ASC associates with NLRP3 on the mitochondrial surface, resulting in inflammasome complex assembly and activation [75]. This represents a novel lipid interaction for caspase-1 with mitochondria having a role as supramolecular organizing centers in the assembly and activation of the NLRP3 inflammasome (Figure 5).

### 2.6. Uric Acid

Uric acid, a product of the metabolic breakdown of purine nucleotides, is usually found in blood close to its solubility limit. As urate concentration increases, uric acid crystal formation, such as monosodium urate (MSU), also increases [76]. Injured cells release uric acid, especially in its crystallized form, working as a DAMP and triggering DCs maturation [77]. Indeed, overproduction of uric acid has been proven to play emerging roles in human disease. MSU is recognized by TLR2 and TLR4 in gouty arthritis [78] and uric acid can stimulate the production of ROS and activate both MAPK and NF-κB pathways [79]. Alongside with gouty arthritis, uric acid has also been associated with the activation of the NLRP3 inflammasome on fibrosis and lung injury inflammation [78]. Several studies demonstrate that uric acid crystals as well as soluble uric acid (sUA) activate NLRP3 inflammasome through lysosomal rupture, accompanied by Ca^2+^ influx [80], and through the production of mtROS, respectively [81].

The role of uric acid in ACD remains yet to be disclosed. Nevertheless, Liu and colleagues showed that MSU enhances the CHS response in BALB/c mice. Indeed, MSU promoted DC maturation and T cell activation, suggesting that it may act as an endogenous adjuvant to potentiate immune response [82].

### 2.7. Cathepsins

Cathepsins are acidic proteases with a key role in cellular protein turnover. Based on their catalytic mechanism, these proteases can be classified as aspartic, serine and cysteine proteases. They have different functions according to their intracellular or extracellular localization but share some redundancy [83,84]. For example, S and L cathepsins, which are cysteine endopeptidases, are involved in MHC II antigen processing and presentation by professional APC [85,86]. Cathepsin B, a lysosomal cysteine endopeptidase, has shown to be involved in T and B cells apoptosis [85] as well as in the proper degradation of immune complexes that bound to the Fcγ receptors on APCs [87]. Furthermore, it plays an important role in inflammatory disorders and antigen presentation mediated by MHC II.

Interestingly, cathepsin B upregulation is dependent on the increase of proinflammatory cytokines [88]. Recently cathepsin B has been implicated in the effector phase of delayed-type hypersensitivity reaction. The topical application of cathepsin B inhibitors before the TNCB challenge caused a significant reduction in ear swelling in the acute cutaneous delayed-type hypersensitivity mouse model. The authors also showed a redundancy in cathepsin function, with cathepsin Z, a protease closely related to cathepsin B, showing a compensatory expression in inflamed ears of cathepsin B-deficient (Ctsb^−/−^) mice. In turn, cathepsin B expression was equally elevated in cathepsin Z-deficient (Ctsz^−/−^) mice [89]. Interestingly, the functional redundancy of cathepsins was also reported by Orlowski and colleagues, which demonstrated that although being involved in IL-1β secretion, cathepsins also regulate pro-IL-1β synthesis [90]. Since NLRP3 inflammasome activation by particulate matter, such as cholesterol crystals or asbestos, was shown to rely on cathepsin leakage from lysosomes, it would be reasonable to hypothesize that sensitizers-induced increase of cathepsin B also contributes to inflammasome assembly [91,92]. However, at least for metal sensitizers, this hypothesis is not verified, since nickel activation of the NLRP3 inflammasome is a phagolysosome-cathepsin B independent process [44].

**Figure 6:**
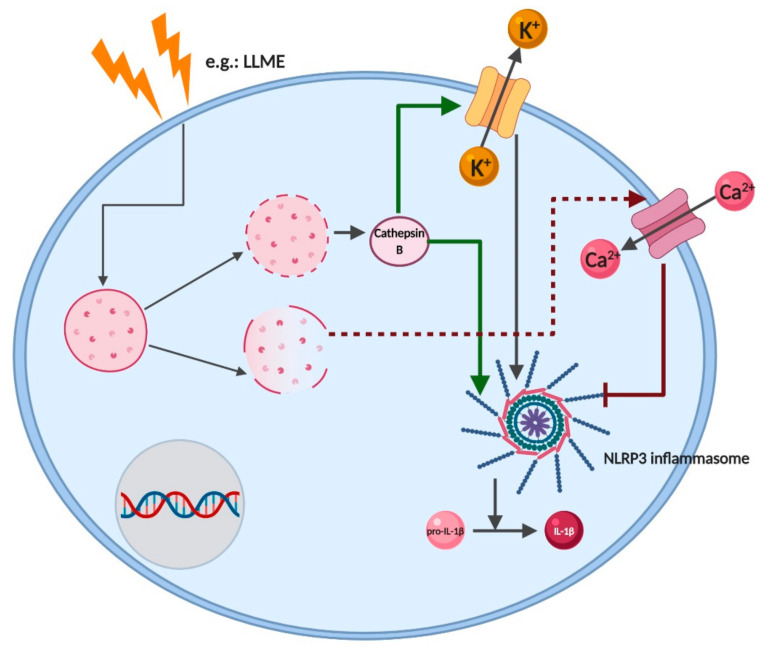
NLRP3 inflammasome activation via cathepsins release. Particulate matter, such as asbestos, are phagocytosed and induce physical destabilization of lysosomal membranes. Slow and partial lysosomal disruption (green arrow) leads to controlled cathepsin B leakage and correlates with K^+^ efflux and robust NLRP3 inflammasome activation. In contrast, an extremely rapid and complete collapse of lysosome integrity (red arrow) correlates with attenuated NLRP3 activation, Ca^2+^ influx and caspase-1-independent necrotic cell death. LLME, Leu-Leu-o-Methyl Ester. Created with BioRender.com.

The link between lysosomal membrane destabilization, cathepsin leakage and inflammasome assembly might be more complex than initially thought. Studies by Katsnelson and colleagues showed that depending on the rate of lysosomal disruption, different outcomes on ions flux and inflammasome activation could be observed [79]. Using the soluble lysosomotropic agent Leu-Leu-o-Methyl Ester (LLME), the authors showed that when lysosomal disruption occurs slowly and in partial increases, leads to K^+^ efflux and NLRP3 inflammasome activation. Conversely, an extremely rapid and complete collapse of lysosome membrane integrity suppressed inflammasome signaling, which correlates with the increased NLRP3 ubiquitination (Figure 6). Furthermore, lysosomal destabilization leads to Ca^2+^ influx which, besides attenuating NLRP3 inflammasome assembly, also potentiates caspase-1-independent necrotic cell death [80].

## 3. Conclusions

NLRP3 inflammasome is a cytosolic structure of uttermost relevance in inflammatory diseases, namely ACD. It is well known that allergens trigger inflammation as well as the release of DAMPs, which can promote the NLRP3 inflammasome activation and ultimately IL-1β secretion. Despite the well-known activators of the NLRP3 inflammasome, studies addressing the connection between skin allergens-induced DAMPs and inflammasome activation, and its relevance for ACD outcome, are still scarce. Inflammasome modulation by skin allergens can occur both at priming and activation steps, through a plethora of DAMPs (Table 1).

The available literature is focused on the involvement of inflammasomes in ACD, although a review about the different DAMPs elicited by the most studied skin sensitizers and their involvement in inflammasome activation is still missing. Indeed, the available studies show that NLRP3 inflammasome assembly by skin allergens is not restricted to one DAMP, instead the plethora of DAMPs elicited by skin allergens work together to induce and sustain inflammasome activation. Therefore, NLRP3 inflammasome can be envisaged as a molecular target for the development of new pharmacological strategies for ACD treatment. Additionally, the elucidation of the key events evoked by skin sensitizers will help to fuel the development of alternative methods to identify skin sensitizers, an important step to replacing animal testing, as demanded by the new European legislation.

## Figures and Tables

**Table 1:** DAMPs elicited by allergens and their involvement on NLRP3 inflammasome priming/activation phases.

Priming Phase		Activation Phase	
DAMPs	Allergens	DAMPs	Allergens
ROS	DNFB, Cr (VI)	mtROS	Cr (VI) Ni
LMWHA	PPD, DNCB	ATP	TNCB
		(ox-)mtDNA	Ni
		Cardiolipin	DNFB
		Uric Acid	TNCB
		Cathepsins	TNCB
